# Intussusception

**Published:** 1880-01

**Authors:** 


### INTUSSUSCEPTION,

IN which one portion of the bowel falls down into another, like as when a stocking is turned half way inside out, effectually prevents any passage from the bowels, and death is inevitable, unless relief is given. Our intimation previously, that a pint of molasses drank while quite warm, had been known to give immediate relief, has caused a suggestion from W. H. Stanton, of Knoxville, that his father's sheep were invariably cured of a similar ailment, by being taken by the hind legs and swung around several times. As ludicrous as this "*operation*" might seem in its application to a human being, we would not hesitate to try it, in case other things failed. To us there is a kind of grandeur in all that is true and practical.

The most violent case of hysterics may be instantly arrested, if a great indignity is offered to the patient, such as slapping in the face with your slipper; and yet that is a better remedy than a plug of the intolerable *Asafœtida*.

